# Consensus on development of a tool to guide implementation of CRPD-aligned standards of universal design and reasonable accommodation for healthcare workers with disabilities

**DOI:** 10.4102/ajod.v15i0.1910

**Published:** 2026-05-29

**Authors:** Meluleki S. Thethwayo, Nomzamo Chemane, Jill Hanass-Hancock, Verusia Chetty

**Affiliations:** 1 Department of Physiotherapy, Faculty of Health Sciences, University of KwaZulu-Natal, Durban, South Africa; 2 South African Medical Research Council, Durban, South Africa

**Keywords:** accessibility, Delphi technique, disability, healthcare workers with disabilities, inclusive, universal design, reasonable accommodation

## Abstract

**Background:**

Despite the existence of South African frameworks guiding the inclusion and accessibility of persons with disabilities in the workplace, there is no tool available to guide how healthcare facilities meet the inclusion and accessibility standards set out in the Convention of the Rights of Persons with Disabilities (CRPD) and national frameworks.

**Objectives:**

This study sought to achieve consensus among experts regarding the tool that can be utilised to guide the implementation of the CRPD-aligned standards of universal design and reasonable accommodation in the public healthcare sector.

**Method:**

A panel of experts (27) in the field of disability across South Africa participated in a two-round modified Delphi technique. The 80% agreement threshold was set to determine the level of agreement among experts.

**Results:**

Consensus was reached on all seven domains (with various disability indicators) as outlined by the CRPD to be included in the development of a tool. These domains are, namely, the prohibition of discrimination, protecting and promoting the rights of persons with disabilities, labour and trade union participation, access to technical and vocational training, employment opportunities and career advancement, reasonable accommodation and professional and vocational rehabilitation training programmes for persons with disabilities.

**Conclusion:**

The panellists agreed that the developed tool can be utilised to evaluate and guide the implementation of universal design and reasonable accommodation in the public healthcare sector.

**Contribution:**

This study provides a consensus-based, CRPD-aligned assessment tool that enables healthcare facilities to systematically evaluate and improve disability inclusion and accessibility in the workplace.

## Introduction

The Constitution of the Republic of South Africa promises human rights to all people, including the right to employment (Republic of South Africa 1996). This means equal access to employment is a right for all people, including persons with disabilities in South Africa. However, it is not enough to guarantee employment for all on paper (including persons with disabilities) but also to ensure that necessary measures are in place to ensure an inclusive and accessible workplace for persons with disabilities in practice.

The White Paper on the Rights of Persons with Disabilities (2016) recognises the rights of persons with disabilities to work on an equal basis with others in the workplace (Department of Social Development 2016). This means that workplaces need to be disability inclusive and accessible in promoting and protecting the rights of persons with disabilities to work on an equal basis with others. The right to fair labour participation is further stipulated in the *South African Employment Equity Act* (Republic of South Africa, 1998) and the *Occupational Health and Safety Act* (Republic of South Africa, 1993), which, respectively, promote equal labour practice and a safe working environment in the workplace.

In achieving the rights of persons with disabilities to work on an equal basis with others, Article 27 of the Convention of the Rights of Persons with Disabilities (CRPD) outlines measures that need to be put in place by state parties in the workplace (United Nations 2006). These include: (1) prohibition of discrimination, (2) provision of reasonable accommodation and universal design, (3) a healthy and safe working environment, (4) access to technical training and (5) access to vocational rehabilitation for persons with disabilities (Article 27). Responding to the CRPD and the White Paper (2016), South Africa has made strides by developing two strategic frameworks, namely, the National Strategic Framework (NSF) on Universal Design and Access (Republic of South Africa, 2021a) and the NSF on Reasonable Accommodation for persons with disabilities (Republic of South Africa, 2021b). These two NSFs guide the employment of persons with disabilities in the workplace by providing national norms for universal design and the provision of reasonable accommodation.

However, despite the existence of these new strategic frameworks and the CRPD, guiding the employment of persons with disabilities in the labour force remains a challenge. There is currently no quality assurance tool available to guide how healthcare facilities are meeting the standards aligned with the CRPD and these South African frameworks. On the contrary, research reveals that healthcare workers with disabilities continue to face barriers around accessibility and inclusiveness in the workplace (Charles, Gie & Musakuro 2023; Govender, Govender & Naidoo 2021; Maja et al. 2011). This is further perpetuated by the lack of guidance on how to meet the needs of healthcare workers with disabilities in the workplace. The absence of a structured monitoring tool makes it difficult for healthcare facilities to assess their compliance with CRPD-aligned standards and identify areas requiring improvement.

Developing a quality assurance tool that assesses the implementation of CRPD-aligned standards of accessibility and inclusion for healthcare workers with disabilities in the workplace would provide healthcare facilities with practical guidance to evaluate accessibility and promote inclusive employment practices and provide better guidance on how to identify and address emerging gaps. Given the complexity of disability inclusion in healthcare facilities and the unavailability of empirical guidance on assessment indicators, expert consensus was required to identify the most relevant disability indicators for such a tool. The Delphi technique is widely used in health research to achieve consensus among experts on complex issues where evidence is limited.

This study forms part of a broader research initiative aimed at developing a validated assessment tool to evaluate compliance with universal design and reasonable accommodation standards for healthcare workers with disabilities in the public healthcare sector. This article reports on expert consensus obtained through a modified Delphi technique to inform the design of such a tool.

## Research methods and design

From July 2025 to September 2025, a panel of 27 experts in the field of disability participated in a two-round modified Delphi technique to obtain consensus on the development of a tool aligned with CRPD standards of universal design and reasonable accommodation for healthcare workers with disabilities in the public healthcare sector.

A modified Delphi approach was employed as the study commenced with a structured questionnaire and a draft assessment tool developed from pre-existing disability assessment tools and the CRPD framework, rather than an open-ended first round typical of a classical Delphi approach. Experts were therefore asked to review, rate and provide feedback on predefined disability indicators. This modification enabled the study to focus on the refinement and validation of indicators aligned with Article 27 of the CRPD and relevant South African disability policy frameworks.

The Delphi technique enabled geographically dispersed experts across South Africa to contribute systematically to the identification and refinement of key indicators for assessing accessibility and inclusion in public healthcare workplaces. The initial questionnaire and draft tool were informed by disability inclusion and accessibility indicators from existing tools developed for healthcare services (Hanass-Hancock et al. 2022, 2025; Otung 2021). Article 27 of the CRPD, together with the NSF on Universal Design and Access (2021b) and the NSF on Reasonable Accommodation (2021a), guided the adaptation of the tool to the workplace context within the healthcare sector.

Purposive sampling was used to recruit participants, and conventional thematic analysis was applied to open-ended responses. Structured communication, anonymity of responses and iterative feedback between rounds were maintained in accordance with established Delphi principles (Barrett & Heale 2020), thereby enhancing methodological rigour. The principal investigator contacted participants via email and distributed the study materials. All responses were consolidated and anonymised to inform revisions to the tool and subsequent rounds of the Delphi process.

### Panel recruitment

A panel of disability experts were purposively recruited following consultation with disability specialists within the researchers’ university and the South African Medical Research Council in KwaZulu-Natal. A maximum variation sampling strategy was used to recruit 20–27 experts in the field of disability in our study to ensure diversity of expertise and perspectives. Inclusion criteria comprised a minimum of 3 years’ experience in the field of disability, involvement in policy or advocacy, relevant academic publications or experience in disability auditing. All 27 experts who were invited met the criteria to participate in our study. All of the 27 experts in the field of disability were part of the panel and voluntarily participated in the first and second rounds of our study. Table 1 reflects the biographical characteristics of our panellists who participated in the study.

**TABLE 1:** Demographic characteristics of participants (*N* = 27).

Variable	Category	*n*	%
Age group (years)	20–30	0	0.0
	31–40	3	11.1
	41–50	10	37.0
	51–60	7	25.9
	> 60	7	25.9
Gender	Male	15	55.6
	Female	12	44.4
Highest qualification	Bachelor’s degree	6	22.2
	Postgraduate certificate (≤ 3 months)	2	7.4
	Postgraduate certificate (≥ 3 months)	2	7.4
	Postgraduate diploma	3	11.1
	Master’s degree	4	14.8
	PhD	10	37.0
Years of professional experience	< 5	2	7.4
	5–10	5	18.5
	11–15	2	7.4
	16–20	3	11.1
	> 20	15	55.6
Years of experience in disability	< 5	1	3.7
	5–10	3	11.1
	11–15	4	14.8
	16–20	2	7.4
	> 20	17	63.0

### Data collection

The researcher developed an initial tool based on available examples and the CRPD Article 27. Using this initial tool, the study worked towards building a consensus with a panel of disability experts through two rounds of the Delphi technique. In the first round, the principal investigator sent an email comprising an information sheet, consent forms, a Delphi questionnaire and an adapted tool to the panel of experts for their review. The experts sent their responses to the researcher via email within 8 weeks.

The researcher analysed the responses from the first round and revised the tool accordingly. The post-Round 1 adaptation process involved modifying disability indicators that did not meet the predefined consensus threshold of 80% agreement.

In preparation for Round 2, anonymised summary results from Round 1, including percentage agreement for each item and a synthesis of qualitative feedback, were shared via email with all 27 participants who completed Round 1. This enabled participants to reconsider their responses in light of group trends, consistent with Delphi methodology. Participants were then invited to review and rate the revised indicators.

Responses from the second round were returned within 2 weeks and analysed by the researchers. A high level of consensus was achieved using an a priori threshold of ≥ 80% agreement. The final version of the tool was subsequently shared with the panel for validation.

Our initial questionnaire tool consisted of seven sections, namely prohibition of discrimination, protecting and promoting the rights of persons with disabilities, labour and trade union participation, access to technical and vocational training, employment opportunities and career advancement, reasonable accommodation, professional and vocational rehabilitation training and return-to-work programmes for persons with disabilities.

These sections emerged from the disability indicators from pre-existing tools (Hanass-Hancock et al. 2022, 2025; Otung 2021) and from the information that emerged during the literature and policy review. Two researchers independently mapped the disability indicators from the pre-existing tools to the Article 27 of the CRPD and yielded seven domains with 37 disability indicators after discrepancies were reconciled. All seven sections required experts to rate the importance of each indicator to be included in the development of a tool on a three-point scale (yes, no and maybe). Open-ended suggestions were added at the end of each section. In addition, the Delphi questionnaire was piloted with two academic researchers who were familiar with Delphi studies and has expertise in the field of disability. This allowed for the modification of the questionnaire and the clarification of ambiguous questions before sending it to the panellists.

### Data analysis

Results from closed-ended questions were analysed using online Google Forms and the Statistical Package for Social Sciences (SPSS), version 27. Furthermore, descriptive statistics, using frequency and statistical distribution, were used for closed-ended questions. The consensus from the panellists was reached with an a priori threshold of 80% agreement, using frequency dispersion on the yes criteria, on the three-point scale. Consensus was defined by a priori as ≥ 80% “yes” endorsement on the three-point scale. Conventional thematic analysis was used to analyse the data from open-ended suggestions. Two independent researchers individually analysed the content from open-ended suggestions to identify common suggestions and the items that needed revision to further modify the questionnaire for the second round. The revised items were incorporated in the second round.

### Ethical considerations

Ethical approval for this study was obtained from the University of KwaZulu-Natal’s Humanities and Social Sciences Research Ethics Committee (Reference: HSSREC/00007773/2024). All fundamental ethical principles guiding research involving human participants were rigorously observed throughout the study.

Panellists were provided with comprehensive information regarding the purpose and procedures of the study, and informed consent was obtained prior to participation. Participation was voluntary. Participants were also made aware of their right to withdraw from the study at any stage without penalty. Anonymity and confidentiality were strictly maintained to ensure the protection of participants’ identities and data integrity.

## Results

A two-round modified Delphi approach yielded a consensus among 27 panels of disability experts. A statistician was consulted to check the reliability analysis of the Delphi survey tool, which showed a Cronbach’s alpha of 0.81 across 37 items, indicating good internal consistency of the instrument. Table 2 reflects the findings of the first round with a frequency distribution of the responses.

**TABLE 2:** Delphi round one responses by section (*N* = 27).

Section or Item	Yes		Maybe		No	
	*n*	%	*n*	%	*n*	%
**A. Prohibition of discrimination**						
Guidelines prohibiting discrimination in recruitment, hiring and career progression	24	88.9	2	7.4	1	3.7
Guidelines promoting equal pay	26	96.3	1	3.7	0	0.0
Guidelines prohibiting harassment	26	96.3	1	3.7	0	0.00
**B. Promotion and protection of rights B1. Safe and healthy working conditions and accessibility**						
Accessible public transport stop within 1 km	26	96.3	0	0.0	1	3.7
Reserved parking bays for persons with disabilities	26	96.3	1	3.7	-	-
Ramps at facility entrances	27	100.0	-	-	-	-
Ramps with compliant slopes	24	88.9	3	11.1	-	-
Steps or ramps with railings and colour contrast	25	92.6	2	7.4	-	-
Doorways and gates wide enough for wheelchairs	27	100	-	-	-	-
Accessible toilet facilities for wheelchair users	27	100	-	-	-	-
Evacuation routes with visual, tactile, auditory alerts	25	92.6	1	3.7	1	3.7
Evacuation routes wheelchair-accessible	26	96.3	-	-	1	3.7
Evacuation routes with warning lights for the Deaf	25	92.6	-	-	-	-
Colour-contrasted doors	26	96.3	1	3.7	-	-
Reception desk suitable for wheelchair users	26	96.3	1	3.7	-	-
Reception area inclusive for diverse disabilities	24	88.9	3	11.1	-	-
Wide passages (≥ 160 cm) free from obstruction	26	96.3	1	3.7	-	-
Tactile and visual way-finding cues	26	96.3	1	3.7	-	-
Non-slip floors	25	92.6	1	3.7	1	3.7
Accessible lifts or all services on ground level	26	96.3	1	3.7	-	-
Doorway space for wheelchair turning radius (≥ 160 cm)	24	88.9	3	11.1	-	-
Good lighting in rooms and corridors	26	96.3	1	3.7	-	-
**B2. Equal opportunities and protection from harassment**						
Disability awareness and sensitisation events	24	88.9	3	11.1	-	-
Anonymous grievance mechanism	20	74.1	5	18.5	2	7.4
**C. Labour and trade union participation**						
Trade unions accommodate persons with disabilities	25	92.6	1	3.7	1	3.7
**D. Technical and vocational training access**						
Reasonable accommodation in workplace training	25	92.6	1	3.7	1	3.7
Budget includes costs for accommodations	27	100	-	-	-	-
Return-to-work support programmes	25	92.6	2	7.4	-	-
**E. Employment opportunities and career advancement**						
To reserve specific posts or vacancies for persons with disabilities and accommodate interview needs	16	59.3	5	18.5	6	22.2
Disability-inclusive platforms for job ads	24	88.9	2	7.4	1	3.7
Traineeships for persons with disabilities	24	88.9	3	11.1	-	-
**F. Reasonable accommodation**						
New staff members to be provided with the needed reasonable accommodation for their workplace no later than 3 months (adjustable desks, reading tablets or magnifiers, large screens)	24	88.9	2	7.4	-	-
Patient files or information in accessible formats (braille, audio, large font)	20	74.1	5	18.5	2	7.4
Temporary assistive devices or technology available	24	88.9	2	7.4	1	3.7
Sign language interpretation available	24	88.9	3	11.1	-	-
**G. Professional and vocational rehabilitation or return-to-work**						
Facility programmes for retention and return to work	26	92.6	1	3.7	-	-
Retraining and accommodations for staff acquiring a disability	25	92.6	2	7.4	-	-

Consensus was reached on all three indicators in section A in the initial survey (prohibition of discrimination in the workplace) supported as crucial to be included in the development of a tool that can be used to guide the implementation of the CRPD-aligned standards of universal design and reasonable accommodation for healthcare workers with disabilities in the workplace. The guidelines promoting equal pay and prohibiting harassment in the workplace both received an overwhelming response of 96.3% from the panel of experts.

In section B, based on the CRPD principles of promoting and protecting human rights in the workplace, there was consensus under the subsection of a healthy and safe working environment and accessibility (subsection B1), with all 19 criteria to be included as the key indicators for inclusion in the tool. Notably, ramps at the facility entrance for the wheelchair users, the facility’s doorways and security gates that are wide enough and independently operable by wheelchair users and toilets that accommodate wheelchair users received 100% consensus from experts. Under equal opportunities and protection from harassment (subsection B2), only one indicator (disability awareness and sensitisation campaigns) yielded a consensus of 88.9% among the panel of experts. Only 74.1% of the panel of experts supported the anonymous grievance mechanism as a crucial indicator for the tool. Following the suggestions and comments from the expert panellists in the first round, the anonymous grievance mechanism indicator was modified to eliminate the ambiguity for the second round.

In the second round, the majority (85.2%) of experts agreed that it is crucial to have systems put in place by the employer for the complaint mechanism for the grievances of employees with disabilities to be redressed.

Under labour and trade union participation (section C), the majority (92.5%) of expert panellists agreed that labour and trade unions must accommodate persons with disabilities in the workplace through the provision of reasonable accommodation and universal design.

In section D (access to technical and vocational training), all three criteria reached consensus to be included as a part of the tool, with the provision of extra cost for reasonable accommodation yielding an overwhelming support of 100% from the expert panellists.

In section E, under employment opportunities and career advancement, only two criteria (disability inclusive platforms for job advertisement and traineeship programmes for persons with disabilities to support career advancement) reached a consensus of 88.9% in the first round. Only 59.3% of the expert panellists supported the reservation of a specific post for persons with disabilities in the workplace as a crucial indicator. Following the suggestions received from the expert panellists in the first round, the reservation of a specific post for persons with disabilities was modified for the second round. In the second round, the majority (96.3%) of the panel of experts agreed that providing equitable and equal opportunities for all, including persons with disabilities, during job advertisement and accommodating interview needs are key indicators for inclusion in the tool.

In section F under reasonable accommodation, out of the four criteria, only three (new staff being provided with reasonable accommodation, provision of assistive devices or technology and availability of sign language interpreters) reached a consensus of 88.9% from the expert panellists. The accessibility of a patient file or information received support of 74.1% in the first round. This resulted in this indicator being modified for the second round based on the suggestions made by the expert panellists. In the second round, 81.5% support was received from the panel for the patient file and information to be in an accessible format for those healthcare workers who are directly managing the patient.

In section G, under vocational and professional rehabilitation and return-to-work programmes, both criteria (programmes for retention and return to work for persons with disabilities and retraining and accommodations for staff acquiring a disability) yielded an overwhelming consensus of 92.6% as key indicators to be included in the tool. Following the content analysis, the open-ended suggestions were used to modify the questionnaire for the second round. Table 3 reflects the responses of the second round for the three items that were revised based on the comments made during the first round.

**TABLE 3:** Summary of disability indicators revised between Delphi Round 1 and Round 2.

Domain	Indicator (Round 1 wording)	Round 1 agreement (%)	Revision made for round 2	Round 2 agreement (%)
Promotion and protection of rights (B2)	Anonymous grievance mechanism for employees with disabilities	74.1	Revised to: Employer must have accessible grievance and complaint mechanisms to address concerns of employees with disabilities	85.2
Employment opportunities and career advancement (E)	Reserve specific posts or vacancies for persons with disabilities and accommodate interview needs	59.3	Revised to: Ensure equitable and accessible recruitment processes, including disability-inclusive job advertisements and reasonable accommodation during interviews	96.3
Reasonable accommodation (F)	Patient files or information available in accessible formats (Braille, audio, large font)	74.1	Revised to: Patient information and files should be available in accessible formats for healthcare workers with disabilities directly involved in patient management	81.5

Following completion of the Delphi process, the final disability inclusion and accessibility assessment tool consisted of seven domains, namely, prohibition of discrimination, protection and promotion of rights, labour and trade union participation, access to technical and vocational training, employment opportunities and career advancement, reasonable accommodation and professional and vocational rehabilitation and return-to-work programmes. These seven domains consisted of 37 disability indicators, reflecting CRPD-aligned standards for inclusive and accessible workplaces.

## Discussion

Our Delphi study aimed at achieving a consensus from the panel of disability experts in developing a tool to guide the implementation of the CRPD-aligned standards of universal design and reasonable accommodation for healthcare workers with disabilities in the public healthcare sector. This tool has the potential to assist healthcare workplaces to meet the standards set out by Article 27 of the CRPD in providing inclusive and accessible workplaces for persons with disabilities. This addresses a long-standing gap in the low employment rate of persons with disabilities in the public healthcare sector.

Importantly, the findings of this study move beyond simply identifying barriers to disability inclusion and instead contribute a structured, consensus-driven framework that operationalises CRPD Article 27 within the healthcare workplace. While previous studies have largely described challenges such as attitudinal and organisational barriers, this study provides actionable indicators that can be systematically assessed and monitored. This represents a critical shift from descriptive evidence towards implementation-focused practice, addressing a key gap in the literature on disability inclusion in healthcare systems, particularly in low- and middle-income contexts.

Despite the progressive policy frameworks in South Africa, existing evidence showed that healthcare workers with disabilities still experience practices of unfair discrimination from their colleagues in the workplace (Charles et al. 2023; Fevre 2017; Govender et al. 2021; Holness 2016; Kontosh et al. 2007; Maja et al. 2011; Okpala 2018). Maja et al. (2011) argue that attitudinal barriers from other colleagues without disabilities towards healthcare workers with disabilities potentially foster discrimination in the workplace. Such behaviour makes healthcare workers with disabilities feel inferior in the workplace as their abilities are often undervalued by their colleagues (Govender et al. 2021; Maja et al. 2011; Narayanan 2018; Waliany 2016). This might be attributable to the lack of guidelines that talk directly to the promotion of rights of healthcare workers with disabilities in the public healthcare sector, covering all the elements of non-discrimination as per Article 27 of CRPD. Research has shown that the lack of reasonable accommodation is not only based on negative attitudes but also a lack of knowledge on how to make provision for persons with disabilities in the healthcare sector (Govender et al. 2021; Hanass-Hancock & Alli 2014; Maja et al. 2011). To mitigate this challenge, the panel of experts in our study agreed that healthcare facilities should have guidelines that spell out how to promote the rights of persons with disabilities during career advancement, hiring, job recruitment, harassment and prohibiting discrimination in terms of remuneration.

Organisational barriers such as physically inaccessible buildings, inaccessible parking and inaccessible working environment are cited as the common theme that prevents persons with disabilities from participating on an equal basis with others in the workplace (Charles et al. 2023; Govender et al. 2021; Maja et al. 2011). Given this challenge, the panel of experts in our study agreed that all healthcare facilities must ensure healthy and safe and accessible working environment for persons with disabilities, through the provisions of disability accommodations such as designated parking for persons with disabilities, provision of ramps leading to all main entrances for the wheelchair users, emergency evacuation routes that are disability inclusive and good lighting conditions. Charles et al. (2023) advocate for management development initiatives for the successful removal of organisational barriers in the workplace, as most managers do not understand the concept of reasonable accommodation and disability. The lack of knowledge about disability among the managers is often attributable to a lack of guidance on disability matters in the workplace (Charles et al. 2023; Govender et al. 2021). This critical gap further contributes to the lack of an inclusive and accessible working environment for healthcare workers with disabilities in the public healthcare sector (Govender et al. 2021). Concurring with the existing evidence, our panel experts supported that disability awareness campaigns and formal guidance are crucial to create an inclusive workplace for healthcare workers with disabilities to work on an equal basis with others (Govender et al. 2021; Majola & Dhunpath 2016).

Evidence shows that persons with disabilities experience barriers to education and vocational training, including in-service training opportunities (Charles et al. 2023; Mutanga 2017; Teborg, Hünefeld & Gerdes 2024).

Our panel of disability experts achieved a consensus that healthcare workers with disabilities should have access to workplace training and development, with a budget allocated for the provision of their specific individual needs during training. In line with the NSF of Reasonable Accommodation for Persons with Disabilities (2021a), our panel of disability experts agreed that the employer needs to have disability budget allocated to cater for reasonable accommodation, and the cost factor should not be a challenge. Hence, budgets are an essential part to break the cycle of a lack of relevant skills for mainstream jobs that is associated with this vulnerable group owing to the disability. On the other hand, Charles et al. (2023) assert that the platforms used for job advertisement are often not disability inclusive or reach out to organisations of persons with disabilities, thus limiting the opportunities of persons with disabilities to participate in the labour force. Our panel of disability experts reached a consensus that employers should use a disability inclusive platform when advertising a job in order to create equal opportunities for healthcare workers with disabilities in the public healthcare sector (Charles et al. 2023). It is further argued by Charles et al. (2023) and Gida and Ortlepp (2007) that the lack of relevant assistive devices and technology in the workplace discourages persons with disabilities from applying for the job and may lead them to leave the workplace as they are not reasonably accommodated. Charles et al. (2023) further explain the challenge posed by managers in the workplace of often viewing the relevant resources required by persons with disabilities to work on an equal basis with others as costly. Hence, our panel of disability experts supported the provision of reasonable accommodations such as assistive devices and sign language interpreters to promote an inclusive and accessible workplace for healthcare workers with disabilities (Mji & Edusei 2019).

There is a paucity of literature about the implementation of vocational and professional rehabilitation to assist persons with newly acquired disabilities to return to work. Our panel of disability experts concurred with Article 27 of the CRPD, that it is crucial for employers to have vocational and professional rehabilitation programmes to promote the retention of those who have acquired disability during employment.

The result of this process has been the completion of a quality assurance tool that now lends itself to further testing to establish whether the tool can be used more widely. This study therefore not only contributes to the conceptual understanding of disability inclusion in healthcare workplaces but also provides a practical mechanism for implementation, bridging the gap between policy and practice.

### Strengths and limitations

Although the Delphi technique ensured broad expert participation, findings are limited by potential self-selection bias and the exclusion of private sector perspectives. Future studies should validate the developed tool through field testing and stakeholder engagement.

### Policy and practice implications

The developed tool has the potential to serve as a national benchmark for assessing inclusivity and accessibility in healthcare workplaces, aligning institutional practices with the CRPD and South Africa’s NSFs.

## Conclusion

This Delphi study achieved expert consensus on indicators essential for developing a tool to evaluate universal design and reasonable accommodation in public healthcare workplaces. The resulting framework addresses a critical gap in South Africa’s disability inclusion monitoring. Future research should focus on piloting and validating the tool across diverse healthcare settings.
